# Association Between Diabetes Mellitus and All-Cause and Cardiovascular Mortality Among Individuals With Ultrasound-Defined Non-Alcoholic Fatty Liver Disease

**DOI:** 10.3389/fendo.2021.773342

**Published:** 2021-12-21

**Authors:** Weiti Wu, Jingjing Xiang, Xiaoye Chen

**Affiliations:** ^1^ Department of Infectious Diseases, Taizhou Hospital, Zhejiang Province, Taizhou, China; ^2^ Department of Emergency, Taizhou Hospital, Zhejiang Province, Taizhou, China; ^3^ Department of Radiotherapy, Taizhou Hospital, Zhejiang Province, Taizhou, China

**Keywords:** non-alcoholic fatty liver disease, diabetes mellitus, NHANES, cardiovascular, mortality

## Abstract

**Objective:**

The influence of diabetes on mortality among patients with non-alcoholic fatty liver disease (NAFLD) in the general population has not been extensively studied. This study aimed to determine the relationship between diabetes and all-cause and cardiovascular mortality in patients with hepatic ultrasound-confirmed NAFLD using data from the Third National Health and Nutrition Examination Survey (NHANES III), 1988–1994.

**Methods:**

Data from 4,037 adult individuals with NAFLD from the NHANES III and mortality outcomes linked to National Death Index records through December 31, 2015, were included. Cox proportional hazards models were used to calculate the hazard ratio (HR) and corresponding 95% CI for mortality from all causes and cardiovascular disease after adjusting for multiple variables.

**Results:**

Among 4,037 subjects with NAFLD (55.9% female), 483 had diabetes at baseline. During a median follow-up of 22.1 years, 1,517 (11.5%) died, including 332 (8.22%) from cardiovascular causes. Diabetes was associated with increased all-cause (HR 3.02 [95% CI 2.67–3.41]) and cardiovascular (HR 3.36 [95% CI 2.61–4.32]) mortality in an unadjusted multivariable Cox regression model. The association remained statistically significant after adjusting for a range of potential confounders (HR 2.20 [95% CI 1.90–2.55] for all-cause mortality and HR 2.47 [95% CI 1.81–3.37] for cardiovascular mortality). An additional stratified analysis did not reveal significantly altered results.

**Conclusion:**

Diabetes was associated with all-cause and cardiovascular mortality in patients with NAFLD. This link could be further characterized in future studies assessing the degree of glycemic control and its relationship with mortality in patients with diabetes and NAFLD.

## Introduction

Non-alcoholic fatty liver disease (NAFLD), defined as the accumulation of hepatic steatosis in ≥5% of hepatocytes without heavy alcohol consumption, is currently the most prevalent chronic liver disease and is one of the leading causes of advanced liver disease worldwide (1, 2). It is currently estimated to affect approximately one-quarter of the global population in the USA (3). NAFLD is a hepatic manifestation of metabolic disease. Among metabolic diseases, diabetes mellitus (DM) has become a major medical problem in the 21st century. Diabetes is a metabolic disorder characterized by increased blood glucose levels following absolute or relative insulin deficiency, β-cell dysfunction, and insulin resistance (4). Such highly prevalent chronic diseases with chronic hyperglycemia are associated with long-term damage to blood vessels and multiple organs. Accumulating evidence has demonstrated that NAFLD is a multi-organ disease, suggesting a strong association between NAFLD and diabetes (5, 6). Diabetes and NAFLD are prevalent diseases that often coexist and can function synergistically to exacerbate the risk of hepatic and extrahepatic clinical outcomes. Recent epidemiological studies have revealed that NAFLD diagnosed using ultrasonography (US) could be used to estimate the development of incident diabetes, and patients with NAFLD have a higher risk of developing diabetes than those without NAFLD (5, 7–9). A recent meta-analysis including 20 studies revealed that NAFLD was related to an increased probability of incident diabetes during a median 5-year follow-up (10). In fact, factors involved in liver fat accumulation generally overlap with risk factors for diabetes. However, there is limited evidence regarding whether diabetes in NAFLD patients over a long period of time enhances the probability of all-cause and cardiovascular mortality.

The Third National Health and Nutrition Examination Survey (NHANES III), conducted between 1988 and 1994, including US data for NAFLD, is a well-designed population-based cohort with a large sample of adults in the USA. In the present study, we aimed to evaluate whether a history of diabetes could affect the risk of all-cause and cardiovascular mortality in patients with NAFLD compared with that in patients with NAFLD but without diabetes by analyzing 27-year follow-up outcome data.

## Materials and Methods

### Study Population and Non-Alcoholic Fatty Liver Disease Definition

A prospective, cross-sectional study using data from NHANES III, a 40-year-old research program sponsored by the USA National Center for Health Statistics, was performed. NHANES III enrolled participants from 1988 to 1994, with data linked to the National Death Index (NDI) up to December 31, 2015. All individuals provided informed consent before participation in the survey, and ethics approval was obtained from the Research Ethics Review Board of the National Center for Health Statistics. All data used and details of the NHANES database are publicly available online (https://www.cdc.gov/nchs/nhanes/about_nhanes.htm); as such, no other local ethics approval was required for the present study. Moreover, detailed research methodologies and protocols are available on the website (https://www.cdc.gov/nchs/nhanes/index.htm). Cross-sectional demographic, socioeconomic, dietary, and medical information were collected through household interviews and clinical and laboratory investigations.

NAFLD is defined as fatty liver identified using US, excluding a competing etiology for secondary liver steatosis, such as heavy alcohol consumption (>1 drinks/day for women or >2 drinks/day for men) or viral hepatitis (defined as laboratory results positive for serum hepatitis B surface antigen or positive serum hepatitis C antibody) (11). Evidence of fatty liver was confirmed according to five standard criteria: liver-to-kidney contrast, bright vessel walls, liver parenchymal brightness, deep beam attenuation, and gallbladder wall definition. The presence of fatty liver was recorded as normal, mild, moderate, and severe hepatic steatosis. The diagnosis of diabetes was based on a positive response to the question “Have you ever been told by a doctor that you have diabetes or sugar diabetes?” (12).

**Figure 1:**
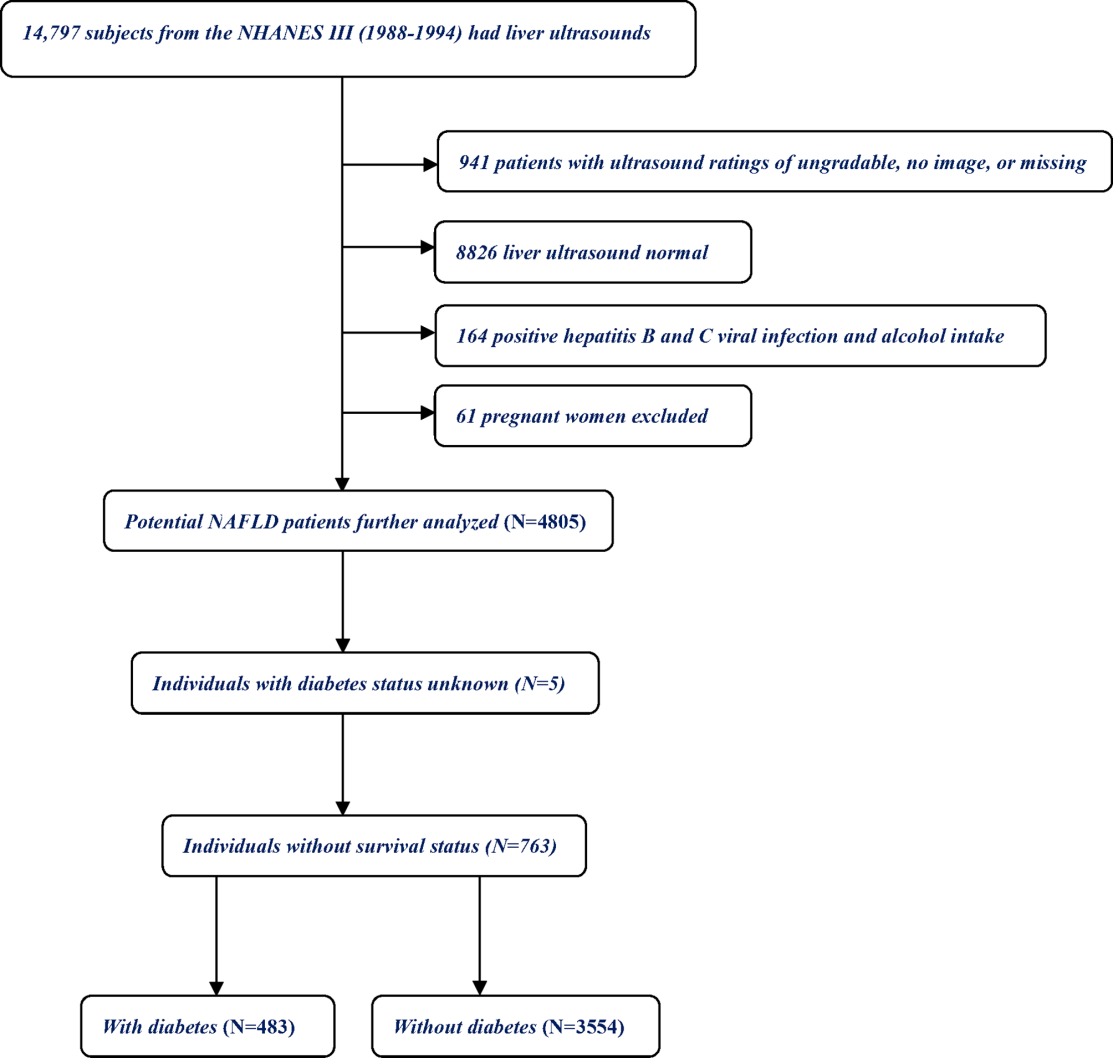
Flowchart of study participants.

Briefly, NHANES III included 14,797 adult individuals who underwent liver US for the diagnosis of fatty liver (**Figure 1**). Participants with normal liver US and those with US ratings of “ungradable”, “no image”, or missing information to determinate fatty liver (n = 9767); those positive for hepatitis B surface antigen and hepatitis C virus antibodies and have heavy alcohol intake (>2 drinks/day for males, >1 drink/day for females); and pregnant women (n = 61) were excluded. Among the remaining 4,805 individuals, those without diabetes status or follow-up data were excluded. Ultimately, the final analysis included 4,037 individuals: 483 with NAFLD and diabetes and 3,554 with NAFLD without diabetes.

### Predictors and Clinical Parameters

Self-reported sociodemographic parameters included age (analyzed as a continuous variable), sex (male or female), marital status (married or unmarried), poverty income ratio, and race (non-Hispanic white, non-Hispanic black, Hispanic, and others). Self-reported medical history included a history of diabetes (yes or no), chronic bronchitis (yes or no), hypertension (yes or no), congestive heart failure (yes or no), stroke (yes or no), and history of cancer (yes or no). Other laboratory variables of interest included albumin, C-reactive protein (CRP), glycosylated hemoglobin (HbA1c), serum cholesterol, triglycerides, high-density lipoprotein cholesterol (HDL-c), glucose, blood urea nitrogen, urinary creatinine, serum creatinine, aspartate aminotransferase (AST), and alanine aminotransferase (ALT). The Fibrosis-4 index (FIB-4) was computed using a previously published algorithm (13), and a FIB-4 index of >2.67 was classified as advanced fibrosis (14). Height, weight, and waist circumference were measured using standard NHANES protocols. Body mass index (BMI) was calculated as body weight (kg) divided by height (m) squared and expressed as kg/m^2^. Because most patients with a history of myocardial infarction could not be identified, this variable was not included in this study considering the sample size.

### All-Cause and Cardiovascular Mortality

Individuals in the NHANES were followed up for mortality by linkage to death records in the NDI. The public-use linked mortality file in the NDI included follow-up duration and underlying cause of death certificate review for adult individuals through December 31, 2015. For individuals who survived after December 31, 2015, the follow-up time was censored. The primary endpoint event in this study was all-cause and cardiovascular mortality. Cardiovascular mortality was defined as any death related to heart disease or cerebrovascular disease, based on the *International Classification of Diseases, 10th Revision* (ICD-10), using the codes I00–09, I11, I13, I20–51, and I60–69.

### Statistical Analysis

Continuous variables with normal distribution are expressed as mean ± SD and as median interquartile range [IQR]) for non-parametric variables, or percentage for categorical variables. Continuous variables between individuals with and without diabetes were compared using *t*-tests (for continuous variables with normal distribution) or non-parametric tests (for non-normal distribution), and categorical variables were compared using chi-squared (χ^2^) statistics. The Kaplan–Meier survival curves were plotted for all-cause and cardiovascular mortality in individuals with NAFLD stratified according to diabetes status. Hazard ratio (HR) and corresponding 95% CI were estimated using multivariable Cox proportional hazards regression to examine the associations between diabetes and all-cause and cardiovascular mortality. Three separate multivariable Cox proportional hazard regression models were used: model 1 was an unadjusted model; model 2 was adjusted for age, sex, BMI, waist circumference, race, and poverty income ratio; and model 3 was the same as model 2 with additional adjustment for education albumin, chronic bronchitis, congestive heart failure, hypertension, stroke, cancer, CRP, HbA1c, cholesterol, triglycerides, HDL-c, glucose, blood urea nitrogen, creatinine, FIB-4, AST, ALT, and urinary creatinine. In addition, stratified analyses across multiple covariates were performed for all-cause and cardiovascular mortality. It has been established that unmeasured confounding is a common difficulty in observational epidemiological studies. Attempts are made to gather data on and to control for as many predictors as possible that are related to the exposure and outcome of interest. Furthermore, we estimated E-values, which were defined as the minimum strength of association that an unmeasured predictor would need to have with both exposure and outcome to completely explain the observed associations between any unmeasured confounder: diabetes (the exposure) and all-cause or cardiovascular mortality (the outcome) (15). E-values range from 1 to infinity, and higher values indicate the need for a higher degree of unmeasured confounding to eliminate the influence of the exposure. Statistical analysis was performed using R software version 3.6.3 (http://www.R-project.org/) and STATA version 14.0 (Stata Corporation, College Station, TX, USA). For all tests, differences with *p*-values of < 0.05 were considered to be statistically significant.

## Results

### Population Characteristics

A total of 4,037 individuals with NAFLD were included in the analyzed cohort, among whom 483 (11.96%) had a history of diabetes at baseline. The baseline characteristics of the cohort are summarized in **Table 1**. Compared with NAFLD individuals without diabetes, those with diabetes were older, more likely to be obese, have a greater waist circumference, and were more likely to be female and Mexican American. Moreover, they were also more likely to have higher FIB-4 and lower AST and ALT levels. There was no difference in the prevalence of other covariates between individuals with and without diabetes. Furthermore, during up to 27.1 years of follow-up (median, 22.1 years), 1,517 (11.5%) NAFLD individuals died from any cause and 332 (8.22%) died from cardiovascular causes.

**Table 1:** Baseline characteristics of the participants by DM in adults aged ≥20 years with NAFLD, NHANES 1988–1994.

Variables	Without DM (N = 3,554)	With DM (N = 483)	*p*-Value
Age (years)	46.73 ± 15.85	57.72 ± 12.76	<0.001
BMI (kg/m^2^)	27.84 ± 6.02	30.41 ± 6.40	<0.001
Waist circumference (cm)	97.62 ± 16.08	94.78 ± 15.26	<0.001
Poverty income ratio	1.80 (0.97–3.17)	1.80 (0.97–3.08)	0.401
Albumin (g/L)	40.87 ± 3.56	40.87 ± 3.49	0.996
CRP (mg/dl)	0.21 (0.21–0.60)	0.21 (0.21–0.55)	0.593
HbA1c (%)	5.80 ± 1.37	5.90 ± 1.64	0.144
Cholesterol (mmol/L)	5.38 ± 1.17	5.42 ± 1.15	0.472
Triglycerides (mmol/L)	1.93 ± 1.67	1.84 ± 1.40	0.309
HDL (mmol/L)	1.27 ± 0.42	1.29 ± 0.44	0.234
Glucose (mmol/L)	6.01 ± 2.59	6.26 ± 3.37	0.056
Blood urea nitrogen (mmol/L)	5.02 ± 1.93	4.88 ± 2.16	0.16
Urinary creatinine (mmol/L)	11.00 (6.20–16.00)	10.50 (5.70–15.62)	0.282
Creatinine (mg/dl)	1.07 ± 0.28	1.05 ± 0.37	0.193
FIB-4	0.87 (0.59–1.21)	1.05 (0.79–1.40)	0.002
AST (U/L)	20.00 (17.00–26.00)	19.00 (16.00–24.00)	0.024
ALT (U/L)	16.00 (11.00–24.00)	15.00 (11.00–22.00)	0.006
Sex (n/%)			<0.001
Female	1,942 (54.64%)	314 (65.01%)	
Male	1,612 (45.36%)	169 (34.99%)	
Race (n/%)			<0.001
Non-Hispanic white	1,300 (36.58%)	138 (28.57%)	
Non-Hispanic black	995 (28.00%)	128 (26.50%)	
Mexican American	1,126 (31.68%)	199 (41.20%)	
Others	133 (3.74%)	18 (3.73%)	
Marital status (n/%)			0.125
Unmarried	1,234 (34.82%)	185 (38.38%)	
Married	2,310 (65.18%)	297 (61.62%)	
Chronic bronchitis (n/%)			0.854
No	3,347 (94.20%)	456 (94.41%)	
Yes	206 (5.80%)	27 (5.59%)	
Congestive heart failure (n/%)			0.279
No	3,415 (96.25%)	460 (95.24%)	
Yes	133 (3.75%)	23 (4.76%)	
Hypertension (n/%)			0.876
No	2,381 (67.49%)	327 (67.84%)	
Yes	1,147 (32.51%)	155 (32.16%)	
Stroke (n/%)			0.103
No	3,465 (97.52%)	463 (96.26%)	
Yes	88 (2.48%)	18 (3.74%)	
Cancer (n/%)			0.086
No	3,358 (94.49%)	447 (92.55%)	
Yes	196 (5.51%)	36 (7.45%)	

DM, diabetes mellitus; NAFLD, non-alcoholic fatty liver disease; NHANES III, Third National Health and Nutrition Examination Survey; CRP, C-reactive protein; HbA1c, glycosylated hemoglobin; HDL, high-density lipoprotein; FIB-4, Fibrosis-4 index; AST, aspartate aminotransferase; ALT, alanine aminotransferase.

### Diabetes and the Risk of Mortality

**Figure 2:**
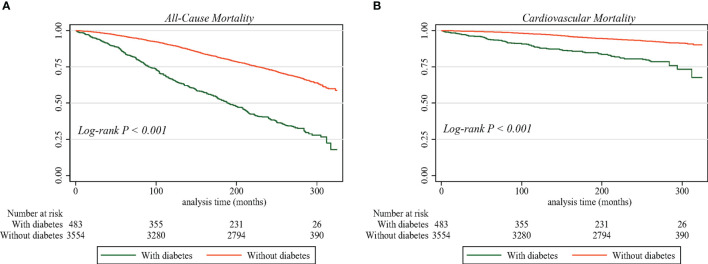
Survival curve by diabetes status in individuals with NAFLD from the NHANES III, 1988–1994. **(A)** All-cause mortality. **(B)** Cardiovascular mortality. NAFLD, non-alcoholic fatty liver disease; NHANES III, Third National Health and Nutrition Examination Survey.

In crude Kaplan–Meier analyses with log-rank test, risks of all-cause (**Figure 2A**) and cardiovascular (**Figure 2B**) mortality were higher among individuals with diabetes than among those without diabetes (all *p* < 0.001). The effect of diabetes and other comorbidities on mortality among individuals with NAFLD was explored using multiple Cox proportional hazards models. As shown in **Table 2**, multivariate Cox regression analysis using the unadjusted model (i.e., model 1) revealed that the HRs for the presence of diabetes among individuals with NAFLD were 3.02 (95% CI 2.67–3.41, *p* < 0.0001) for all-cause mortality and 3.36 (95% CI 2.61–4.32, *p* < 0.0001) for cardiovascular mortality as compared with participants without NAFLD. After age, sex, BMI, waist circumference, race, and poverty income ratio (i.e., model 2) were adjusted, individuals with diabetes still demonstrated a higher risk of death, with an HR of 2.14 (95% CI 1.86–2.46) for all-cause mortality and 2.22 (95% CI 1.65–2.97) for cardiovascular mortality. Moreover, after further adjustment for all potential confounders (i.e., model 3), diabetes remained associated with an increased risk of both all-cause and cardiovascular mortality, with an HR of 2.20 (95% CI 1.90–2.55) and 2.47 (95% CI 1.81–3.37), respectively.

**Table 2:** Hazard ratio for all-cause mortality and cardiovascular mortality by Cox proportional hazards models.

All cause and cardiovascular mortality	Hazard ratio (95% CI)	*p*-Value
All causes mortality		
Model 1	3.02 (2.67, 3.41)	<0.0001
Model 2	2.14 (1.86, 2.46)	<0.0001
Model 3	2.20 (1.90, 2.55)	<0.0001
Cardiovascular mortality		
Model 1	3.36 (2.61, 4.32)	<0.0001
Model 2	2.22 (1.65, 2.97)	<0.0001
Model 3	2.47 (1.81, 3.37)	<0.0001

Model 1 was adjusted for none. Model 2 was adjusted for age, sex, BMI, waist circumference, race, and poverty income ratio. Model 3 was adjusted for age, sex, BMI, waist circumference, race, poverty income ratio, albumin, chronic bronchitis, congestive heart failure, hypertension, stroke, cancer, CRP, HbA1c, cholesterol, triglycerides, HDL, glucose, blood urea nitrogen, creatinine, FIB-4, AST, ALT, and urinary creatinine.

BMI, body mass index; CRP, C-reactive protein; HbA1c, glycosylated hemoglobin; HDL, high-density lipoprotein; FIB-4, Fibrosis-4 index; AST, aspartate aminotransferase; ALT, alanine aminotransferase.

### Stratification Analyses and Sensitivity Analyses

**Figure 3:**
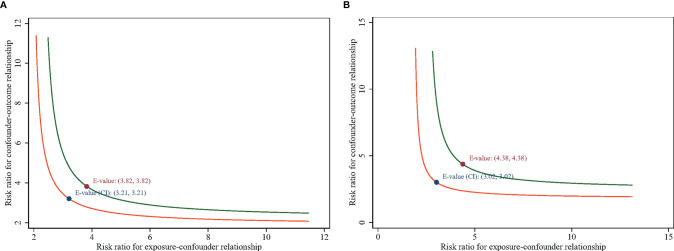
E-value for the lower 95% CI and point estimate in **(A)** all-cause mortality and **(B)** cardiovascular mortality.

In stratification analyses, consistent results were obtained when analyses were stratified according to sex, race, marital status, history of chronic bronchitis, congestive heart failure, hypertension, stroke, cancer, and with and without advanced fibrosis, which was determined according to FIB-4. No significant changes were detected between mortality and these stratified variables after adjustment for age, BMI, waist circumference, poverty income ratio, albumin, CRP, HbA1c, cholesterol, triglycerides, HDL-c, glucose, blood urea nitrogen, creatinine, FIB-4 (continuous variable), AST, ALT, and urinary creatinine (**Table 3**). In this model, advanced fibrosis was also an independent risk factor for all-cause mortality. In addition, an E-value sensitivity analysis was performed to measure the robustness of the association with unmeasured confounding factors. The E-value (and the lower limit of its 95% CI) for the association between diabetes and all-cause mortality was 3.82 (3.21), whereas that for the association of diabetes with cardiovascular mortality was 4.38 (3.02). For unmeasured confounders associated with diabetes and all-cause mortality during the follow-up, the E-value equation yielded a value of 3.82 (**Figure 3A**). The result could be interpreted as an unmeasured confounder that was associated with both diabetes and all-cause mortality by an HR of 3.82-fold each, above and beyond the measured confounders; in contrast, weaker confounding would not do so. Furthermore, diabetes and cardiovascular mortality had an HR of at least 4.38 (**Figure 3B**) beyond the measured confounders, but not by weaker confounding (the corresponding lower limit was at least 3.02). The higher the E-value, the stronger the confounder association must explain the exposure and outcome away. As such, the E-value provided evidence supporting the robustness of the study.

**Table 3:** Stratification analyses of the association of DM with all-cause mortality and cardiovascular mortality in individuals with NAFLD.

	All-cause mortality			Cardiovascular mortality		
	N	HR (95% CI)	*p*-Value	N	HR (95% CI)	*p*-Value
Sex						
Female	2,256	2.03 (1.66, 2.48)	<0.0001	2,256	2.09 (1.32, 3.29)	0.0015
Male	1,781	2.34 (1.90, 2.88)	<0.0001	1,781	2.56 (1.70, 3.86)	<0.0001
Race						
Non-Hispanic white	1,438	2.71 (2.12, 3.46)	<0.0001	1,438	2.62 (1.60, 4.27)	0.0001
Non-Hispanic black	1,123	2.34 (1.77, 3.09)	<0.0001	1,123	2.30 (1.22, 4.33)	0.0099
Mexican American	1,325	1.98 (1.52, 2.58)	<0.0001	1,325	2.05 (1.17, 3.59)	0.0123
Others	151	1.02 (0.32, 3.31)	0.9676	151	NA	NA
Marital status						
Unmarried	1,419	2.15 (1.69, 2.74)	<0.0001	1,419	2.15 (1.26, 3.65)	0.005
Married	2,607	2.13 (1.78, 2.55)	<0.0001	2,607	2.44 (1.68, 3.56)	<0.0001
Chronic bronchitis						
No	3,803	2.12 (1.83, 2.46)	<0.0001	3,803	2.17 (1.58, 2.98)	<0.0001
Yes	233	3.97 (1.93, 8.15)	0.0002	233	20.23 (4.43, 92.37)	0.0001
Congestive heart failure						
No	3,875	2.16 (1.87, 2.51)	<0.0001	3,875	2.33 (1.71, 3.19)	<0.0001
Yes	156	4.98 (1.61, 15.39)	0.0053	156	NA	NA
Hypertension						
No	2,708	2.08 (1.74, 2.49)	<0.0001	2,708	2.19 (1.49, 3.23)	<0.0001
Yes	1302	2.19 (1.71, 2.81)	<0.0001	1,302	2.51 (1.50, 4.19)	0.0004
Stroke						
No	3,928	2.18 (1.88, 2.51)	<0.0001	3,928	2.36 (1.73, 3.20)	<0.0001
Yes	106	2.20 (0.36, 13.31)	0.3903	106	NA	NA
Cancer						
No	3,805	2.26 (1.95, 2.62)	<0.0001	3,805	2.51 (1.84, 3.42)	<0.0001
Yes	232	1.61 (0.80, 3.26)	0.1815	232	0.86 (0.12, 6.26)	0.882
FIB-4						
≤2.67	3,765	2.13 (1.84, 2.46)	<0.0001	3,765	2.22 (1.63, 3.02)	<0.0001
>2.67	65	8.51 (1.94, 37.28)	0.0045	65	NA	NA

Adjusted for age, BMI, waist circumference, poverty income ratio, albumin, CRP, HbA1c, cholesterol, triglycerides, HDL, glucose, blood urea nitrogen, creatinine, FIB-4 (continuous variable), AST, ALT, and urinary creatinine.

DM, diabetes mellitus; NAFLD, non-alcoholic fatty liver disease; BMI, body mass index; CRP, C-reactive protein; HbA1c, glycosylated hemoglobin; HDL, high-density lipoprotein; FIB-4, Fibrosis-4 index; AST, aspartate aminotransferase; ALT, alanine aminotransferase.

## Discussion

In this nationally representative cohort study, we aimed to evaluate the impact of diabetes on mortality risk in individuals with NAFLD. We found that a history of diabetes was significantly associated with a higher risk of all-cause and cardiovascular mortality in individuals with NAFLD. These associations were independent of sociodemographic factors, medical history, laboratory investigation indicators, and degree of liver fibrosis. Furthermore, these results remained unchanged in stratification analyses. Stratified analysis according to sex, race, marital status, chronic bronchitis, congestive heart failure, hypertension, stroke, cancer, and degree of liver fibrosis confirmed that NAFLD with diabetes significantly increased the risk of all-cause and cardiovascular mortality in the age, BMI, waist circumference, poverty income ratio, albumin, CRP, HbA1c, cholesterol, triglycerides, HDL-c, glucose, blood urea nitrogen, creatinine, FIB-4 (continuous variable), AST, ALT, and urinary creatinine-adjusted model as compared with individuals without diabetes. An important potential source of bias in the observational epidemiological analysis is residual confounding. However, calculating the E-value supported the robustness of the main findings.

DM and NAFLD share common pathophysiological mechanisms, including insulin resistance and chronic inflammation (16–18). The link between diabetes and NAFLD is reflected by a series of metabolic changes, such as insulin resistance, triglyceride metabolism, and defective hepatic lipid profile, which results in fat accumulation, immune responses, and hyperinsulinemia induced by β-cell dysfunction in individuals with diabetes (19). Insulin resistance has been reported in 66%–83% of patients with NAFLD (20). NAFLD and insulin resistance share some common predisposing genetic variations, such as Apo C3 (16, 17). Hepatic insulin clearance is suppressed in individuals with diabetes and is related to the severity of metabolic syndrome (21). Insulin has anti- and pro-inflammatory properties (22). Moreover, a recent study revealed that serum insulin levels were strongly correlated with ballooning and hepatic lobular inflammation (23).

Several epidemiological studies have reported an association between diabetes and NAFLD. Recent evidence supports a more complex link between diabetes and NAFLD than was previously observed, exhibiting a bidirectional and mutual association between them (24). NAFLD has been reported to be a risk factor for type 2 DM (T2DM), and T2DM is a risk factor for liver disease in patients with NAFLD (6, 25, 26). A survey performed in Scotland revealed that, if a diabetic individual had ever been discharged with a hospital diagnosis of NAFLD, there was a greater risk of cardiovascular (HR 1.70 [95% CI 1.52–1.90]) and all-cause (HR 1.60 [95% CI 1.40–1.83]) mortality compared with those free of NAFLD (27). A recent study revealed that advanced liver fibrosis, measured using hepatic transient elastography, is a risk biomarker for cardiovascular mortality in individuals with T2DM and NAFLD. The prevalence of diabetes in individuals with NAFLD depends on the degree of NAFLD, ranging from 9.8% in mild NAFLD to 17.8% in moderate-to-severe NAFLD (28–30). NAFLD is reported as an independent risk factor for the development of diabetes, with a two-fold increase in incidence in these patients, and participants with non-alcoholic steatohepatitis have a more than three-fold higher risk of developing incident diabetes than those with simple steatosis (10, 28). A previous study included 132 patients with liver biopsy-confirmed NAFLD, and 44 (33%) had a history of diabetes (30). After multiple potential confounders (age, BMI, and cirrhosis) were adjusted, all-cause mortality was greater in patients with NAFLD and diabetes (relative risk 3.30 [95% CI 1.76–6.18]). The risk of liver-related mortality was higher in patients with NAFLD and diabetes. However, the main shortcoming was the selection bias introduced by the inclusion of individuals from a tertiary care center. In fact, patients undergoing liver biopsy demonstrated a higher HR for mortality, despite the fact that this was unaffected by age, sex, or date of diabetes diagnosis (31). This highlights the selection bias of non-community-based studies that depend on biopsy and the challenge in generalizing findings from participants who have undergone liver biopsy or liver US among the general population in the community. Furthermore, participants who undergo biopsy are more likely to have serious liver disease and, thus, exhibit a greater risk of liver-related mortality. Therefore, these studies overestimate mortality rates when used in the general community. Our study was based on a nationally representative cohort that can be generalized to the general population.

This mechanism between diabetes and NAFLD is complex and involves not only the liver but also the immune system, skeletal and cardiac muscle, brown and white adipose tissue, pancreas, vascular system, gut, and brain (32). The link between diabetes and NAFLD can be described by a spectrum of metabolic changes represented by insulin resistance, defective hepatic lipid profile, and triglyceride metabolism, which result in fat accumulation, immune responses, and hyperinsulinemia, as determined by β-cell dysfunction in T2DM (19). The link between diabetes and NAFLD further confirmed that diabetes aggravates the course of NAFLD and that, conversely, NAFLD will result in metabolic decompensation of diabetes (33). Another possible mechanism may be that ectopic liver fat is perhaps part of the pathogenic process in diabetes, resulting in hepatic insulin resistance, excess gluconeogenesis, and higher fasting glucose levels (34). Moreover, hepatic steatosis due to NAFLD leads to increased aminotransferase levels, with ALT levels exceeding those of AST (35). Increased ALT levels are prevalent in patients with T2DM, and for a specific serum ALT, those with T2DM exhibit more liver fat than those without diabetes (36).

The present study has several limitations, the first of which was that the identification of NAFLD status relied on a retrospective chart review of liver US results without biopsy evaluation/confirmation. However, liver biopsy is unethical for epidemiology studies of asymptomatic individuals in the NHANES III. It is the most comprehensive population-based database with ultrasound-controlled attenuation parameters to confirm hepatic steatosis using well-defined criteria (37). US is presently the most commonly available and non-invasive technique to diagnose hepatic steatosis in clinical practice. The sensitivity of US may vary depending on the hepatic fat content; when implemented properly, US has been uncovered to identify as little as ≥5% hepatic fat content (38). A meta-analysis that included 49 studies has displayed high accuracy of US (area under the curve (AUC) value of 0.93) for the diagnosis of moderate-to-severe steatosis (39). Second, due to the observational nature of the study, causation cannot be determined. Third, several variables were self-reported, which may have resulted in recall and reporting biases. Third, individuals were only queried about diabetes at baseline at a single time point; as such, any time-varying effect could not be investigated. Future *in vitro* and *in vivo* experiments should aim to elucidate the molecular mechanisms by which diabetes is associated with the prognosis of patients with NAFLD.

Notwithstanding these limitations, this study had several strengths. First, we used nationwide survey data representing the U.S. general population, and mortality data were linked to the NDI of the National Center for Health Statistics with a long follow-up period to observe mortality events, and ICD-10 codes were used to identify the cause of death. Second, the prospective design with a relatively large sample size and the use of a nationally representative sample of USA individuals with NAFLD facilitated generalization of the conclusions. Furthermore, several stratification analyses and calculations of E-values support the robustness of the findings.

## Conclusion

In a large cohort of adults with NAFLD, we found that diabetes was significantly associated with higher all-cause and cardiovascular mortality. This link could be further characterized in future studies assessing the degree of glycemic control and its relationship with mortality in patients with T2DM and NAFLD.

## Data Availability Statement

Publicly available datasets were analyzed in this study. The raw data used in the article are available from National Health and Nutrition Examination Survey program (https://www.cdc.gov/nchs/nhanes/index.htm).

## Author Contributions

WW and XC are the principal investigators. WW and JX conducted the formal statistical analysis and data management. WW and JX wrote and XC edited and revised the manuscript. All authors read and approved the final manuscript.

## Conflict of Interest

The authors declare that the research was conducted in the absence of any commercial or financial relationships that could be construed as a potential conflict of interest.

## Publisher’s Note

All claims expressed in this article are solely those of the authors and do not necessarily represent those of their affiliated organizations, or those of the publisher, the editors and the reviewers. Any product that may be evaluated in this article, or claim that may be made by its manufacturer, is not guaranteed or endorsed by the publisher.
